# Synergies, Discrepancies, and Action Priorities: A Statewide Engagement Study to Strengthen Clinical Research in Cerebral Palsy

**DOI:** 10.1111/hex.70257

**Published:** 2025-04-24

**Authors:** Melissa M. Murphy, Gavin T. Colquitt, Paige S. Ryals, Katie Shin, William C. Kjeldsen, Allison McIntyre, Sydni V. W. Whitten, Christopher M. Modlesky, Nathalie L. Maitre

**Affiliations:** ^1^ Emory University School of Medicine Atlanta Georgia USA; ^2^ Appalachian State University Boone North Carolina USA; ^3^ University of Georgia Athens Georgia USA; ^4^ Children's Healthcare of Atlanta Atlanta Georgia USA

**Keywords:** cerebral palsy, community‐based participatory research, research partners, trainee pathways

## Abstract

**Background:**

Cerebral palsy (CP) clinical research is fraught with challenges, in part due to health‐related disparities common among people with disabilities. Perspectives of people with lived experience of CP, clinicians and researchers vary on how to address these disparities. The present initiative explores synergies and discrepancies among stakeholders (*n* = 212) representing these partner groups in perceived barriers and facilitators to high‐quality clinical CP research and robust trainee pathways. The overarching goal is to generate priority actions to empower meaningful partner group engagement in CP research and, ultimately, improve health outcomes for people with CP.

**Methods:**

Grounded in empowerment theory, mixed methods needs assessments were conducted separately with partner groups to capture perspectives on barriers and facilitators to high‐quality CP research and strong trainee pathways. Thematic analysis was applied to focus groups and interviews to identify themes and subthemes.

**Results:**

Discrepancies among partner groups emerged related to informational needs, community connection, ethical research and equitable representation in research, and fair compensation for lived experience partner engagement in the research process.

**Conclusions:**

Ongoing opportunities for researcher action to empower partner group engagement include building shared purpose, nurturing social connection within and among groups and intentional efforts to build trust and codesign studies.

**Patient or Public Contribution:**

The initiative described here was informed by caregivers of children with CP from Georgia, USA, using a community‐based participatory research (CBPR) approach. CPBR is a collaborative approach, designed to give communities, which here include people with lived experience of CP, control over research processes and outcomes. Their perspectives were essential to the premise of this study and guided data interpretation, especially with regard to how their perspectives may or may not correspond to those of CP researchers and clinicians. To ensure inclusion of all perspectives, individuals with CP were also represented in these latter two engagement groups. Finally, the design, conduct, analysis and interpretation of data were informed by a researcher and a clinician‐scientist, both of whom have lived experience as caregivers of children with CP.

**Abbreviations**
CBPR: community‐based participatory research
CP: cerebral palsy
GA: Georgia, USA
NIH: National Institutes of Health
PAR: participatory action research
US: United States

## 1 Introduction

Research to advance the health of people with cerebral palsy (CP) has been supported by varied US organisations and has recently been highlighted in national initiatives in the United States [1, 2]. However, clinical research in people with CP is fraught with challenges, many originating from disparities common to those with disabilities and especially pronounced for people who experience intersectional discrimination and inequities (e.g., race, gender and socio‐economic status) [3]. Varied perspectives among invested groups exist on how to best address disparities that pervade the health and lives of individuals, and consequently, clinical research: *patients and their caregivers* have lived experiences of barriers and facilitators to positive health outcomes and participation, *clinicians* have knowledge of approaches to improve health outcomes and *researchers* have training in research strategies and methods to answer questions that can guide clinical decision making. To that end, the present initiative sought to explore and describe the state of CP research from these different perspectives. The overarching goal was to identify areas of synergy and discrepancy in barriers and facilitators to clinical research in CP, which could then drive an action plan to strengthen clinical research deemed critical for improving health outcomes.

CP is the most common lifespan physical disability originating in childhood and affects 3/1000 live births [4, 5] and ~1 million people in the United States [6, 7]. CP refers to a phenotypic spectrum that results from variable non‐progressive insults in the perinatal period, leading to movement disorders and multiple secondary conditions such as hip dislocation, balance issues and hand dysfunction that limit independence and severely impact quality of life [8]. A decade ago, lifelong expenses related to having a child with CP (medical bills, education, social services and lost wages) financially impacted families for a total of $1 million per person with CP [9]—equivalent to ~ $1.3 million adjusted for inflation. Maternal health, perinatal factors, pregnancy complications and healthcare disparities all increase risks of CP [10]. High‐poverty states have poorer maternal health and access to preventative care, and concurrently, higher concentrations of people with disabilities, especially those with mobility limitations [11]. These states present a unique context for CP research, due to lifespan contributions of adverse social determinants of health and perinatal disparities. Thus, the present exploration describes a comprehensive endeavour to understand and strengthen CP clinical research in one of these US states.

In the present initiative, we focused on two priority areas for US research: clinical research and workforce development [1]. By virtue of their training and experience, researchers are best qualified to share perspectives on the state of research. However, as a group, they cannot directly speak to the perspectives and experiences of other invested groups—including people and families with lived experience of CP (referred to as *lived experience partners*), clinicians, trainees and training programme administrators (referred to as *workplace partners*). Therefore, engaging these partner groups is critical to meaningful and successful clinical research.

Substantial progress has been made to establish a patient‐centred agenda for CP clinical research [12]; however, more work must address racial disparities in CP research, diversity among trainees, multidisciplinary teams, community‐engaged research and shared research tools and resources [2]. These areas are interrelated, and each is multifaceted. Developing and implementing lasting solutions addressing these priority challenges will require understanding the perspective of people with lived experience of CP, researchers, clinicians, allied health trainees and trainee programme administrators.

The present study represents the first implementation step (exploration) of a strategic effort to listen to these groups, with the goal of identifying actionable steps and priorities to strengthen CP research and training, and ultimately maximise lived experience partners' engagement in CP research.

To achieve this objective, we initially engaged researchers to assess the perceived landscape of CP research within the state of Georgia, USA. From 2017 to 2020, Georgia saw increases in maternal mortality, an indicator of overall maternal health, from 25.1 to 30.2 per 100,000 live births, with mortality rates per 100,000 live births among non‐Hispanic, Black mothers (48.6) more than double the rate among non‐Hispanic, White mothers (23.3) [13]. Focusing on a single state allows for a more detailed exploration of region‐specific barriers and facilitators to CP research, such as state‐level policies, healthcare infrastructure and community resources. Additionally, this targeted approach helps identify unique local challenges while generating insights that can be applied to improve CP research and care strategies at a broader regional or national level. Based upon themes that emerged from researcher responses, we then asked each partner group for their separate perspectives on barriers and facilitators. Finally, using overarching themes, we generated priority actions to empower meaningful involvement of CP lived experience and workplace partners in research.

## 2 Materials and Methods

### 2.1 Underlying Theoretical Framework

Empowerment theory is one approach for engaging and empowering marginalised populations to achieve sustained change [14]. Empowerment is defined as a community‐centred process where individuals gain control over their lives, participate democratically in their community and critically understand their environment [15, 16]. An ‘empowered state’ requires group participation and dialogue to enhance individual belief in change and growth while diminishing power imbalances [17].

Participatory action research (PAR) is a methodology grounded in empowerment theory and successfully used in CP research [18]. PAR allows the creation of dynamic networks whereby participants take ownership of problems and work collaboratively to solve complex problems that are deeply meaningful to their larger community [19]. As discussed subsequently, data collection for this initiative included World Café‐style focus groups [20]. A form of PAR, the World Café approach is objective, structured and self‐directed while suspending existing power dynamics and ensuring democratic contributions [19]. It gives communities control over research processes and outcomes and is well‐aligned with empowerment theory [21]. The present initiative uses this approach for researcher and caregiver focus groups (Table S1).

### 2.2 Design

A series of mixed methods needs assessments were conducted with partner groups to engage them as equal partners in the co‐creation of programming and research initiatives to improve the state of CP research and strengthen professional pathways. Quantitative surveys (caregivers and clinicians), focus groups (caregivers, researchers and trainees) and semi‐structured interviews (programme administrators) were conducted to address the following: (1) considerations when deciding whether to participate in a research study (caregivers) and (2) barriers and facilitators of (a) caregiver participation in research (researchers), (b) clinician collaboration in research (researchers, clinicians), (c) building and maintaining pathways for entering or remaining in the field of CP professionals (researchers, clinicians, trainees and programme administrators).

### 2.3 Ethics

This project was designed for quality improvement purposes. The researcher project was deemed as ‘not research involving human subjects’ by the University of Georgia Human Subjects Office (PROJECT00007325). Similarly, the other projects were recognised as quality improvement initiatives and so deemed as not ‘human subjects research’ by the Emory University Institutional Review Board. Data were collected via graphical recording and analysed in aggregate. No identifiers were associated with individual responses.

### 2.4 Setting

Caregiver, researcher, clinician and trainee perspectives were obtained from focus groups that took place at CP‐focused events from April 2023 to July 2023 (described below). Programme administrator perspectives were collected during individually scheduled interviews between November 2023 and January 2024.

#### 2.4.1 2023 Cerebral Palsy Foundation Early CP Health Summit (Clinicians and Caregivers)

This implementation‐focused summit focused on birth‐3 years brings together more than 300 professionals across disciplines including neonatology, neurology, paediatrics, physical and occupational therapy, early intervention and nursing with the goal of accelerating translation of early detection and intervention knowledge into practice. In 2023, the Summit launched a ‘parent track’ to connect and empower caregivers to support and advocate for their young child with CP.

#### 2.4.2 2023 Research Symposium on the State of CP Research (Researchers)

This regional research‐focused conference brought together more than 60 researchers, including US National Institutes of Health (NIH) funded researchers and clinician‐scientists, university administrators, trainees and students, and clinician‐scientists with an interest in advancing CP research in Georgia, USA.

#### 2.4.3 Early Detection of CP Trainings (Clinicians)

Clinicians from Georgia's five regional high‐risk infant follow‐up clinics received intensive in‐person training on assessments for early detection of CP through a series of site‐specific workshops colead by the senior author (N.L.M.).

#### 2.4.4 CP Summer CAMP Programme (Trainees)

Three sessions of this week‐long, intensive, therapy‐guided CAMP programme for young children (12–36 months) were held between June and July 2023. The CAMP was staffed by students and allied health trainees working with a trained clinical and research team.

### 2.5 Eligibility Criteria and Recruitment

Attendees at the CP‐focused events were invited to participate, including caregivers of a child with CP, basic science and applied researchers with a research interest in CP, medical or allied health clinicians (e.g., occupational, physical, music and speech therapy, social work, and kinesiology), and research or clinical students and trainees in these disciplines. Programme administrators in university departments overseeing internship experiences and placements for trainees participating in the CP‐focused events were identified and invited by project team members.

### 2.6 Participants

Table 1 presents participant demographic characteristics by partner group.

**Table 1:** Demographic characteristics of CP researcher,^a^ caregiver, and trainee stakeholder groups.

	Number (%)
*Researcher* (*N* = 66)	
Current level	
Student/trainee	25 (38%)
Faculty/researcher	16 (24%)
Therapist/clinician	16 (24%)
Community partner	1 (2%)
Professional affiliation	
University within Georgia	48 (73%)
Georgia Northeast Regional Education Service Agency	13 (20%)
University outside of Georgia^b^	4 (6%)
Professionals who self‐identified as having cerebral palsy	2 (3%)
*Caregiver characteristics* (*N* = 19)	
Age (years)	
< 20	1 (5%)
21–30	3 (16%)
31–50	13 (68%)
60+	2 (10%)
Relationship to child with cerebral palsy	
Mother	14 (74%)
Father	2 (10%)
Other family member	2 (10%)
Other legal guardian	1 (5%)
Education level	
< 7th grade	1 (5%)
7–9th grade	1 (5%)
High school graduation	2 (10%)
Partial college or trade school	2 (10%)
College graduation	5 (26%)
Graduate education	8 (42%)
Child characteristics	
Less than 1 year	4 (21%)
1–3 years	15 (79%)
Race	
Black or African American	8 (42%)
White	11 (58%)
Ethnicity (% not Hispanic)	18 (95%)
*Trainee* (*N* = 17)	
Area of study	
Allied health (e.g., occupational or physical therapy, speech language pathology and social work)	8 (47%)
Biology/neuroscience/pre‐medicine	6 (35%)
Engineering	2 (12%)
Kinesiology	1 (6%)
Level of study	
Undergraduate	11 (65%)
Graduate	3 (18%)
Doctoral	1 (6%)
Post doctoral	2 (12%)
University affiliation	
Public (University of GA, GA Tech, GA State and GA Southern)	9 (53%)
Private (Emory, Brenau)	8 (47%)
Race	
Black	6 (35%)
Asian	5 (30%)
White	5 (29%)
Latino	1 (6%)
*Clinician* (*N* = 102)	
Profession	
Therapist (music, occupational, physical and speech language pathology)	66 (65%)
Medical doctor	22 (22%)
Nurse/nurse practitioner	10 (9%)
Other	4 (4%)
Clinician recent/primary position	
Academic hospital or medical system (in and out‐patient)	50 (49%)
Academic‐affiliated private hospital, medical system, or practice (in and out‐patient)	32 (31%)
Other	20 (20%)
Position geographical region	
Southeastern United States	51 (50%)
Midwestern United States	22 (21%)
Northwestern United States	12 (12%)
Northeastern United States	9 (9%)
Southwestern United States	3 (3%)
Outside North America	3 (3%)
Central/Mideastern United States	2 (2%)
Length in position	
0–5 years	24 (24%)
5–10 years	25 (25%)
10–20 years	24 (23%)
20+ years	28 (27%)
Prefer no answer	1 (1%)
Gender	
Female	90 (88%)
Male	11 (11%)
Non‐binary	1 (1%)
Age (years)	
21–30	21 (20%)
31–50	53 (52%)
51–60	13 (13%)
60+	13 (13%)
Prefer no answer	2 (2%)
Special access requirements/ADA accommodations	
Yes	1 (1%)
No	99 (97%)
Prefer no answer	2 (2%)

^a^ Sixty‐six people attended the conference, though not all respondents participated in answering all questions for the needs assessment; two attendees facilitated the needs assessment (M.M.M. and S.W.).

^b^ Southeastern United States (*n* = 2), Midwestern United States (*n* = 2).

### 2.7 Data Collection

#### 2.7.1 Quantitative (Clinician)

A project‐designed survey was administered to clinicians to minimise time burden associated with participation (see Appendix A). Survey data were collected and managed using REDCap (Research Electronic Data Capture), a secure, web‐based software platform [22, 23] hosted at Emory University. Surveys took about 10 min to complete. Basic demographics were collected. Respondents used a sliding scale from 0 (*not at all*) to 100 (*very much so*) to rate the importance of facilitators and barriers in two areas: CP researcher‐clinician collaborations and sustaining CP‐focused trainee development pathways.

#### 2.7.2 Qualitative (Caregiver, Researcher, Trainee and Programme Administrator)

*Focus groups*. After a welcome and overview of the required democratic processes necessary for participation, a host was selected at each table to facilitate discussion, encourage all voices to be heard and act as lead table representative during whole group discussion [24]. Discussion took place over a series of ‘rounds’ guided by prompts eliciting discussion of barriers and facilitators to engagement (Tables S2–S4). All participants were encouraged to share insights and collaboratively note vital concepts and emerging connections [19].

Discussion rounds in the Café were followed by ‘whole‐group harvesting’ [19] where each table shared their responses with the group at large. The two facilitators employed the evaluation tool of graphic recording [25] to visibly compile data from the whole group discussion, facilitate consensus building within the larger group and provide the opportunity for additional discussion, clarification and comments. This process allowed for built‐in member checking to ensure valid responses from all participants [26], via a member check of synthesised data [27] using a process of real‐time member checking to increase data validity [28]. Facilitators also made field notes used in conjunction with graphic recordings to clarify ambiguous responses. Responses from graphic recordings and field notes were transcribed into Excel, breaking them down by row into individual responses and checked for accuracy by authors (M.M.M., P.S.R.). Response clarifications were noted within brackets in the transcription.

*Structured interviews (programme administrators)*. In total, 20–30‐min interviews were conducted via Zoom Workplace with programme administrators representing allied health training programmes (*n* = 8) of trainees through a range of public and private institutions in Georgia, USA, including Georgia Southern Exercise Science, Brenau Occupational Therapy, Georgia State Occupational Therapy, Physical Therapy, Public Health, and Social Work, University of Georgia Music Therapy and University of West Georgia Speech Language Pathology. Specific questions guided discussions and follow‐up questions clarified and expanded responses (Table S4). Audio transcriptions from cloud recordings were reviewed by a project team member for transcription accuracy. Responses were parsed according to the target question addressed and then added to Excel for coding.

### 2.8 Analysis

#### 2.8.1 Quantitative

Quantitative data from surveys were analysed using SPSS version 29 [29]. Descriptive statistics are reported as frequencies, means and standard deviations. Medians and ranges are presented for variables where the data distribution is skewed.

#### 2.8.2 Qualitative

We used an inductive reasoning approach [30] and constant comparison to explore key concepts emerging from the responses [31]. Following an individual review of the data, team members (M.M.M., P.S.R., W.K., and K.S.) worked in pairs to identify initial recurring themes and associated subthemes for each partner group. This classification was done by focusing on the meaning expressed (i.e., ‘What is this statement about?’). Responses were then sorted and summarised for each group.

To ensure intra‐rater reliability of initial thematic coding, responses were coded a second time at a later date. All coding disagreements were discussed and resolved. In cases where agreement was below 80% [32, 33], original responses and code categories were revisited and further clarified or collapsed. Reliability testing was then repeated to ensure Cohen's Kappa was > 0.80 for all partner groups [32].

Once reliability was established within partner groups, team members (M.M.M., P.S.R., and K.S.) reviewed themes and subthemes across groups to arrive at a comprehensive set of themes and subthemes reflective of all partner perspectives [34]. When necessary, adjustments were made to theme labels to ensure consistent terminology without compromising the original meaning of the category. To ensure robustness and validity of the final themes, responses from partner groups were cross‐verified and referenced against source data. Once theme triangulation was complete, intra‐rater reliability was repeated for theme agreement (% agreement = 91.6%, Cohen's Kappa = 0.91) and subtheme agreement (% agreement = 94.04%, Cohen's Kappas ≥ 93%). Team members (M.M.M., P.S.R., and K.S.) then examined discrepancies and synergies in subthemes among partner groups.

*Discrepancies* were noted when a similar theme/subtheme was expressed differently by one or more groups. *Synergies* occurred when groups were matched in their characterisation of a theme/subtheme. Within subthemes, responses could refer to a *barrier*, defined as a real or potential obstacle to CP research (e.g., a lack of something) or a *facilitator*, including an existing resource or construct that made it easier for CP research to flourish (e.g., the presence of something). Facilitators could also include improvements to overcome common barriers frequently observed in CP research. This dual role of barriers and potential facilitators is consistent with findings of a recent review of participation in clinical trials by people with disabilities [35].

## 3 Results

From open‐ended partner responses, priority areas for building and sustaining strong CP research efforts were characterised by four overarching themes: involvement, connection, funding and research approach. Tables 2, 3, 4, 5 summarise themes and subthemes. Illustrative examples from partners are in Tables S5–S8. For simplicity, results that follow focus on synergies and discrepancies among partner responses within each of the four overarching themes.

**Table 2:** Definition of *involvement* theme and subthemes among CP research partner groups with areas of synergy (‘S’) and discrepancy (‘D’) indicated.

Involvement		
Encouraging other stakeholder groups to participate in research and/or incorporating other stakeholder groups into research (one‐directional communication, emphasising transactional participation rather than relationship), including four subthemes		
S	Awareness of opportunities	Communicate broadly about current research studies and promote opportunities to participate in research.
S	Logistical burden to access opportunities	Logistical details, such as distance, cost and timing, that may inform decisions to participate in research (caregivers) or decision to pursue internship or training opportunity (trainees).
S	Attitudes and perspectives^a^	Subjective reasons related to perceived interest in CP research (interest) or inherent qualities of mind or character (disposition).
D	Informational needs	**Researcher:** Researcher perceived need for families to have ***information about general topics*** (e.g., CP‐specific knowledge, benefits of research, parenting strategies and study dissemination). Researchers perceived needs of clinicians to have information about active studies and study results.
		**Caregiver:** ***Specific benefits to family/child, information about their child***, quality/qualifications of research team, results of studies and when/how they will have access to information from research.
		**Trainee/programme admin:** ***Information about specialty area/content***, scientific/research methods.
		**Clinicians** ^b^ **:** Information about active studies, summaries of completed study findings, research designs and benefits of research for clinical care.

*Note:* ***Bold*** text indicates discrepancies in subtheme characterisation among groups.

^a^ Noted only among non‐caregiver groups.

^b^ Based upon clinician ratings of helpfulness derived from researcher open‐ended responses; ratings confirm clinician‐researcher synergy rather than discrepancy.

**Table 3:** Definition of *connection* theme and subthemes among CP research partner groups with areas of synergy (‘S’) and discrepancy (‘D’) indicated.

Connection		
Communication or collaboration with stakeholder groups (bidirectional communication emphasising relationship rather than participation), including six subthemes		
S	Professional community connection and development^a^	Communication pathways to increase connections among professional groups (e.g., networking, professional development events)
S	Interdisciplinary and interinstitutional collaborations^a^	Building partnerships and sharing resources across disciplines and institutions. May also refer to international collaborations/relationships or opportunities/experiences gained from collaborations, which can include research and clinical work or clinical applications.
S	Educational and/or training initiatives^a^	Formal programmatic opportunities to train current and future professionals (e.g., continuing education credits, training programmes and internships) and/or opportunities to engage with patients/families.
—	Involvement of K‐12 partnerships^b^	Building and promoting formal collaborations with K‐12 schools and educational professionals.
D	CP community connection	**Researcher:** ***Researcher initiated interaction with the community*** intended to communicate about research, promote parent‐to‐parent connection, involve community in programmes.
		**Caregiver:** Interest in ***connecting with other families***, especially those who have participated in similar studies.
		**Trainee/programme admin/clinicians:** Not observed.
D	Pathways for bidirectional communication among stakeholders	**Researcher:** ***Partnering with patients/families in all stages of research process*** and setting research priorities; ensuring patient/family perspectives are understood and research endeavours are meaningful.
		**Caregiver:** ***Not observed***.
		**Trainee/programme admin** *: **Communication pathways to increase connections within and among stakeholder groups*** (e.g., conferences, clinician‐family partnerships).
		**Clinician:** Including clinicians as an active part of the research team, study development.^c^

*Note:* ***Bold*** text indicates discrepancies in subtheme characterisation among groups.

^a^ Noted only among non‐caregiver groups.

^b^ Noted only among researcher group.

^c^ Based upon clinician ratings of helpfulness derived from researcher open‐ended responses; ratings confirm clinician‐researcher synergy rather than discrepancy.

**Table 4:** Definition of *research approach* theme and subthemes among CP research partner groups with areas of synergy (‘S’) and discrepancy (‘D’) indicated.

Research approach		
How to do better research, recruitment, retention, study design, registry and so forth and including seven subthemes		
S	Participant experience during research recruitment/participation	Emphasis on aspects of study participation that make up the ‘experience of participation’—from recruitment to completion.
D	Ethical research and equitable representation in research	Researcher: Refers to adherence to principles: respect for persons, beneficence and justices. Reference can be to presence of trust or lack of trust/need to build trust in research or research meeting needs of community; historical and systemic barriers, socio‐economic barriers and other disparities that affect access to researcher or its benefits.
		Caregiver: Not observed.
		Trainee/programme admin: Not observed.
		Clinician: Not rated.
—	Research design and/or process	Refers to study design elements, use of technology or aspects of conducting research (e.g., team science and working with others); can include design/process related to use or access to registry/research.
—	Study information data elements	Specific information about study opportunities to include in a registry.
—	Quality control	Ensuring the quality of the data elements within a registry.
—	Potential participant data elements	Specific information about participants to be collected by a registry.
—	Strategies for increasing recruitment	Options for outreach to promote study opportunities and enrolment.

Clinicians rated importance and facilitators/barriers in two areas based upon themes derived from initial thematic analysis of researcher responses: CP researcher‐clinician collaborations and sustaining CP‐focused trainee development pathways (Tables 6 and 7, respectively). For ease of comparison, their quantitative responses are incorporated into results from the thematic analysis (Tables 2, 3, 4, 5). Of note, the high degree of variability in ratings likely reflects differences across institutions and researcher collaborators.

**Table 5:** Definition of *funding* theme and subthemes among CP research partner groups with areas of synergy (‘S’) and discrepancy (‘D’) indicated.

Funding		
Financial needs related to research including three subthemes		
S	Clinician/trainee support^a^	Financial support for students (e.g., paid internships) or clinicians (e.g., compensation for clinician time and/or protected time).
—	Research process or infrastructure^b^	Funding support to build and sustain research programmes; funding not otherwise specified.
D	Family compensation	**Researcher:** ***Providing payments to participants*** to offset costs of time, travel and other costs of participating in research.
		**Caregiver:** ***Not observed***. Compensation‐related responses aligned with logistics subtheme.
		**Trainee/programme admin:** Not observed.

*Note:* ***Bold*** text indicates discrepancies in subtheme characterisation among groups.

^a^ Noted only among non‐caregiver groups.

^b^ Noted only among researcher group.

**Table 6:** Clinician ratings of possible facilitators of researcher‐clinician collaborations: Scale 0–100.

	Median rating (range)
‘Practices’ that could allow more effective CP researcher‐clinician collaborations	100 = *very helpful*
Provide updates to clinicians in the format of short briefs when a research study has been completed^a^	85 (29–100)
Create platform that hosts study updates for completed studies^a^	78 (20–100)
Create platform that hosts active studies recruiting participants^a^	80 (19–100)
Host learning experiences for newly published research (e.g., CEU courses, grand rounds)^b^	84 (0–100)
Include clinicians as an active part of the research team in performing tests, assessments and/or interventions^b^	82 (21–100)
Involve CP clinicians more in the development of a research study or aim^b^	76 (34–100)
Design more research studies that seamlessly integrate within standard clinical care processes^c^	84 (40–100)
Compensate clinician effort on research studies^d^	81 (30–100)
Possible barriers	100 = *very much a barrier*
No access to information about research studies being conducted^a^	65 (0–100)
Not interested in being involved in research activities^a^	50 (0–100)
Do not know enough about research designs or its benefits for clinical care^a^	50 (0–100)
No access to CP researchers^b^	67 (0–100)
Not have enough training to participate effectively in research activities^b^	61 (0–100)
Not have enough time to participate in research activities^d^	85 (0–100)
Not paid enough to participate in research activities^d^	70 (0–100)
Perceived implementation of ‘practices’ to help researchers and clinical practitioners collaborate.	100 = *very helpful*
Can easily refer a patient to actively recruiting studies^a^	50 (0–100)
Are trained about comparative effectiveness research and benefits for clinical care^a^	34 (0–100)
Regularly receive updates (e.g., study result summary) from researchers when studies have been completed^a^	26 (0–100)
Play an active role in the administration of interventions and/or assessments of research studies^b^	(0–100)
Play an active role in the development of research studies^b^	50 (0–100)
Are compensated for their time engaged in research studies^d^	25 (0–100)

*Note: n* = 102; main themes are ^a^involvement, ^b^connection, ^c^research approach and ^d^funding.

**Table 7:** Clinician ratings of possible facilitators of trainee pathways: Scale 0–100.

	Median rating (range)
Possible facilitators	100 = *very important*
Clear and consistent communication from programme administration about training expectations^a^	85 (34–100)
Mentors within the field^b^	90 (31–100)
Trainee programmes that engage or promote relationships across institutions^b^	84 (27–100)
Exposure to multiple pathways or areas of interest throughout training^b^	90 (50–100)
Help/ways to fund their training^d^	90 (25–100)
Possible barriers	100 = *very much a barrier*
Trainees are not aware of opportunities to work with patients with CP^a^	50 (0–100)
Perceived complexity of treating patients with CP^a^	50 (0–100)
Perceived time commitment of this training^a^	67 (0–100)
Trainees do not have access to education/training in treating patients with CP^b,c^	55 (0–100)
Training is too expensive^d^	73 (0–100)
Frequency of actions of the practice/institution to maintain pathways for upcoming clinicians who treat CP	100 = *very helpful*
Clear and consistent communication from programme administration regarding expectations of training^a^	50 (0–100)
Trainees are provided mentors within the field^b^	50 (0–100)
Relationships with other institutions and programmes^b^	50 (0–100)
Trainees are exposed to multiple pathways or areas of interest throughout training^b,c^	56 (0–100)
Trainees are provided help/ways to fund their training^d^	50 (0–100)

*Note: n* = 85; main themes are ^a^involvement, ^b^connection, ^c^research approach and ^d^funding.

### 3.1 Theme 1: Involvement

Discussion prompts queried barriers and facilitators of participation, engagement or collaboration in research (Tables S3 and S4). *Involvement*‐related responses to these prompts reflected acknowledgement of uni‐directional (e.g., researchers to caregivers) information flow rather than bidirectional communication (e.g., researcher and caregiver dialogue) to encourage participation in research (Table 2).

#### 3.1.1 Synergies

Responses across partner groups converged on three important aspects of involvement in CP research: awareness of opportunities to participate in research (caregivers)/CP‐focused training experiences (trainees, clinicians), logistical barriers to access opportunities (e.g., cost, timing and distance) and attitudes about CP research.

#### 3.1.2 Discrepancies

A fourth subtheme, *informational needs* to participate in research, also emerged across groups. However, differences emerged in how groups discussed these needs. Researcher responses focused on general informational needs, such as CP‐specific knowledge/education, broad benefits of research participation and ‘parent education.’ Caregiver responses, however, focused on specific informational needs, such as the direct benefit to their child/family, the qualifications of the team conducting the research, their child's individual results and/or development and how they can quickly access results from the study they participated in. At the same time, informational needs of trainees and programme administrators reflected specialty area‐specific information and educational needs (e.g., ‘ensuring students understand the scientific method…’).

Similar to trainees, clinician respondents rated informational needs, including short summaries of completed research studies and descriptions of active studies as somewhat to very helpful and lack of information about the benefits of research for clinical care as a barrier. And yet, as a whole, they perceive summary updates (Med = 26, 0–100) as lacking at their institutions (Table 6).

### 3.2 Theme 2: Connection

Connection‐related responses to the discussion prompts captured an acknowledgement of, or desire for, collaboration with and among groups beyond unidimensional informational or transactional involvement in research efforts (Table 3).

#### 3.2.1 Synergies

Many subthemes within connection reflected synergistic assessment of formal and informal CP partner relationships among trainees and professionals (rather than lived experience partners). For example, the need for interdisciplinary, professional and institutional collaborations was described by researchers, trainees and programme administrators and reflects the importance these partner groups place on building pathways to increase connections and partnerships among CP‐professionals. Although variable, clinicians also rated educational initiatives as very helpful (Med = 84, 0–100; Table 6) to facilitate researcher‐clinician collaborations and the lack of such initiatives as a barrier (Med = 61, 0–100; Table 6).

#### 3.2.2 Discrepancies

Two subthemes within connection, however, reflect discrepancies in how connection is envisioned among partner groups: *CP community connection* and *pathways for bidirectional communication among partners*. From a researcher's perspective, CP community connection broadly encompassed researcher‐initiated interactions with the CP community intended to communicate with families, connect parents together and ‘increase…[non‐CP community's] exposure to individuals with CP.’ Caregiver responses were more specific in considering research participation as a means to connect to the CP community, especially other families that have participated in similar studies (e.g., ‘other parents are the best resources’).

The second subtheme where discrepancies were noted, *pathways for bidirectional communication*, was articulated by researchers as partnering with patients/families in all stages of the research process. None of the caregivers' responses were coded with this subtheme despite being questioned similarly. Responses from trainee and programme administrators did express the need to increase connections within and among partner groups noting a ‘lack of infrastructure/pathways…to continue communication and involvement in CP Community and research.’

For their part, clinicians rated opportunities to be included as part of the research team (Med = 82, 21–100) and involvement in study development as somewhat or very helpful (Med = 76, 34–100; Table 6). Although variable, as a whole, they perceive inclusion on research teams (Med = 50, 0–100) and involvement in study development (Med = 50, 0–100) to be ‘implemented’ but leaving room for improvement (Table 6).

### 3.3 Theme 3: Research Approach

Research approach‐related responses articulated how research is conducted, including study design, recruitment, retention, etc. (Table 4). No trainee or programme administrator responses consistent with this theme were noted. Clinicians were not asked to rate perceptions of the research approach.

#### 3.3.1 Synergies

Researcher and lived experience partner responses acknowledged participant experience during research as an important consideration for participation. Participant experiences included the overall satisfaction and trust in the research team, accessible settings, welcoming environment, convenient timing and location, and direct and clear communication.

#### 3.3.2 Discrepancies

Researcher responses articulated a subtheme of ethical research and equitable representation in research that was not directly articulated by the other partner groups. In their responses within this subtheme, researchers acknowledged (1) historical and systemic barriers, socio‐economic barriers and other disparities that affect trust in research or the ability of research to meet the needs of the community (e.g., ‘equipment that perpetuates disparities (e.g., EEG caps that require wetting hair [for a study in a predominantly Black/African American community])’, ‘[find ways to include people who are nonspeaking]’, ‘protect people in registries from those who would use them or their data in less beneficial ways’) and (2) the need for more pathways for people with CP to become professionals in the field of CP (e.g., ‘more people with CP should do research’).

### 3.4 Theme 4: Funding

Funding‐related responses captured financial needs associated with research (Table 5). No caregiver responses consistent with this theme were noted, such as compensation for participation on advisory boards or panels. However, the cost of research participation (e.g., transportation, time away from work) was identified as a logistical issue for participation.

#### 3.4.1 Synergies

Responses from researchers, trainees, programme administrators and clinicians converge on the importance of providing financial support for trainee and clinician effort in research. For example, clinicians rated trainee financial support as somewhat of a barrier (lack of) and very much a facilitator (help with) to strong trainee pathways for entering or remaining in the field of CP.

In reference to themselves, clinicians rated ‘compensate clinical effort on research studies’ as very helpful for facilitating research‐clinician collaborations (Med = 81, 30–100; Table 6), and lack of protected time for research activities as very much a barrier (Med = 85, 0–100; Table 6) and lack of compensation somewhat a barrier (Med = 75, 0–100; Table 6). And yet, when asked about the perceived implementation of this practice, respondent ratings indicated that compensating clinicians for their time engaged in research studies was not very well implemented (Med = 25, 0–100; Table 6).

#### 3.4.2 Discrepancies

Researcher responses articulated the importance of providing compensation to families engaged in research to offset costs associated with participation as part of the research process (e.g., lived experience advisory board participation) and study participation. Although caregivers did not specifically express responses aligned with this subtheme, they acknowledged financial considerations for participation. However, their responses were best classified under the logistics subtheme of Involvement (e.g., ‘length, frequency, location, cost [of participating]’), as one of several factors contributing to participation decisions rather than a factor that offsets participation.

## 4 Discussion

**Figure 1:**
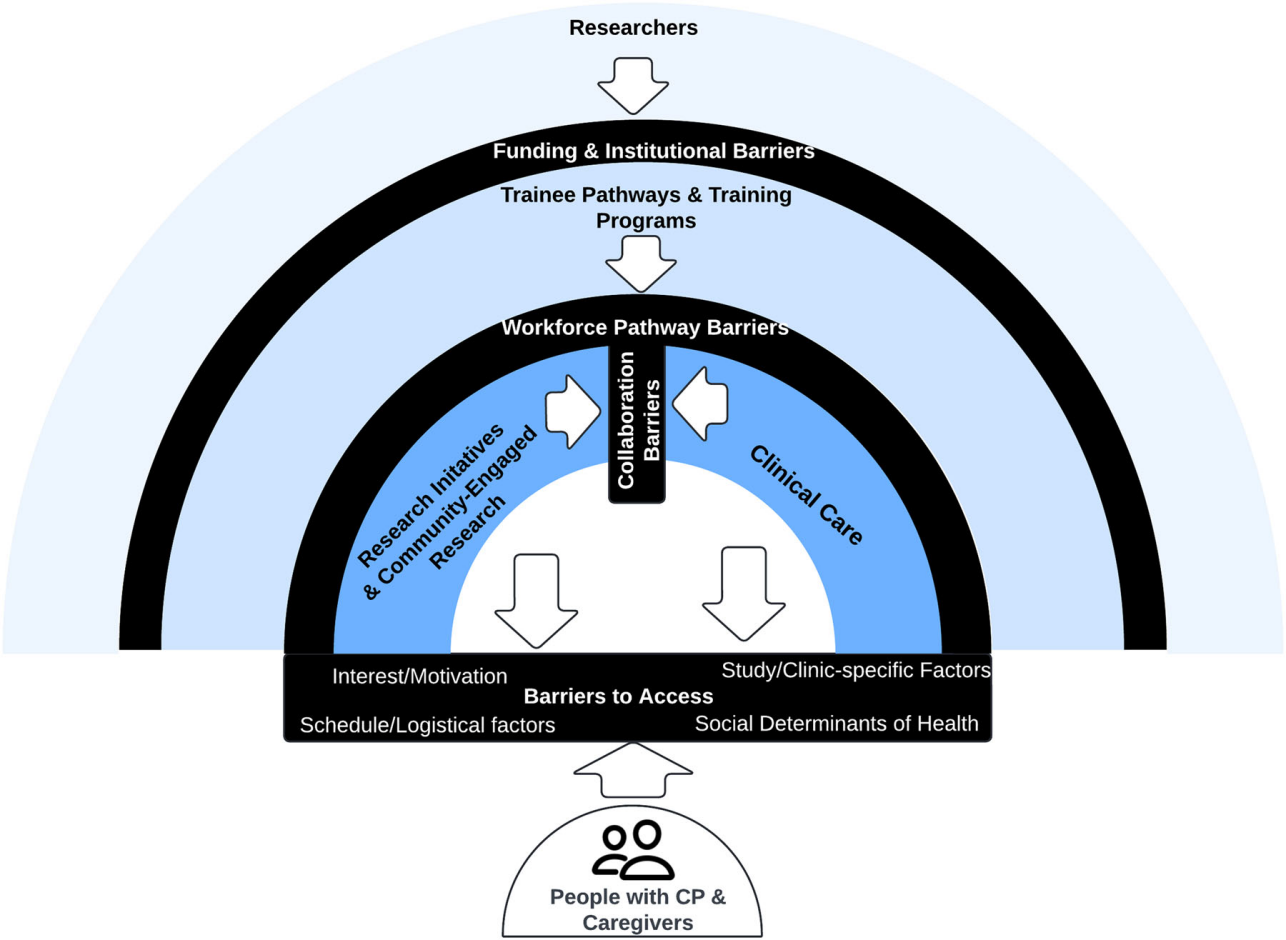
Bio‐ecological framework for understanding barriers to research participation in Georgia, USA. Barriers to people with CP and caregivers being at the centre of the model exist at all levels. Barriers reflect communication difficulties between levels, insufficient integration of clinical and research initiatives, and limited inclusion of trainees and training programmes in integrated model of clinical research.

A mixed methodology approach was used to engage five partner groups in constructive dialogue to improve the research landscape and ultimately improve the lives of people with CP in Georgia [24]. To depict the complexity of perspectives and factors that inform the quality and context of CP research, findings are situated within an ecological systems framework (Figure 1). This framework also provides a structure to guide systematic efforts to eliminate barriers to and enhance facilitators of research participation.

**Figure 2:**
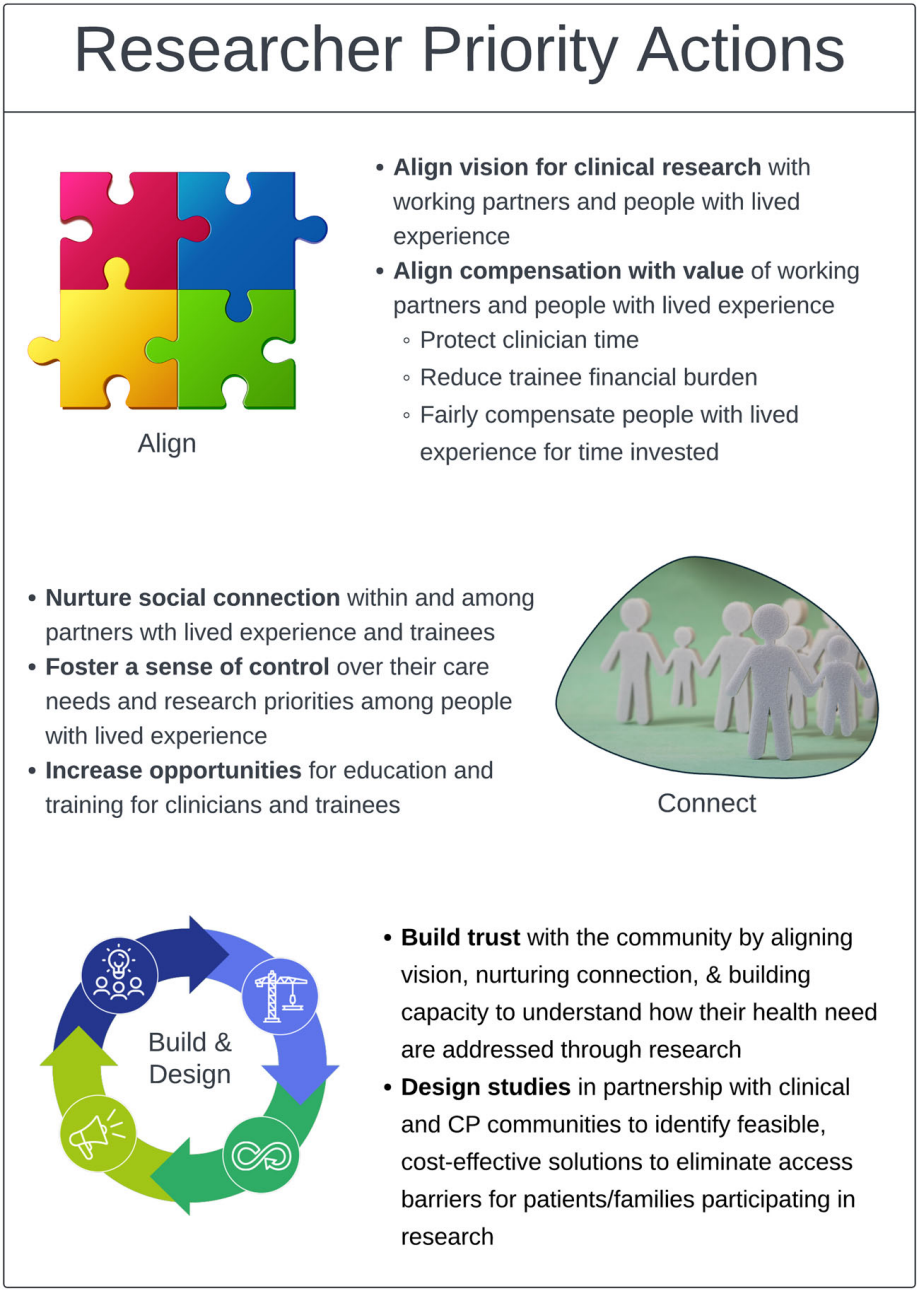
Researcher priority actions for engaging CP partners in research.

Barriers to CP research in Georgia currently exist at all levels of the socio‐cultural context in which research takes place (e.g., research initiatives, clinical care and training programmes). A range of health determinants (e.g., biological, behavioural and environmental) prevent people with CP and their caregivers from being at the centre of a fully integrated model of clinical research. Barriers identified in the present initiative, such as study burden, lack of information and distrust of researchers, are consistent with those documented in other studies [36]. Similarly, facilitators identified in the present initiative, such as compensation, strong relationships, diversity sensitivity and culturally sensitive communications, are also consistent with other studies [37]. Together, these findings suggest that while some progress has been made, continued growth is needed to communicate the benefits of research, improve access and more effectively connect with the CP community. Three areas of ongoing opportunity for researcher action exist (Figure 2).

### 4.1 Researcher Priority Actions

#### 4.1.1 Align Purpose

To leverage synergies and reconcile differences, CP partner groups must work together towards a common purpose. Multiple studies have already identified shared CP research priorities [12, 38, 39, 40]. Although not the only factor contributing to effective team outcomes [41], shared purpose in interprofessional healthcare teams improves team effectiveness [42, 43], ability to identify and resolve problems among team members [44], innovation [41, 43] and high‐quality care for patients [45].

In the absence of shared purpose, team members may experience a sense of isolation—especially minority team members [41]. Feelings of isolation and loneliness are associated with a lack of *social connection* [46, 47, 48]. Fostering shared purpose and nurturing social connections may be especially important for encouraging research participation—especially among lived experience research partners, who may represent a minority perspective on research teams dominated by basic science researchers and clinician‐scientists.

Researchers can make intentional and systematic efforts to build shared purpose by seeking to understand healthcare needs and associated research priorities of lived experience partners and aligning research programmes with shared priorities that bring direct and tangible benefits. Community‐based participatory research (CBPR) approaches, including PAR like those used in this initiative, are a strategy‐rich source of community‐accessible methods, including toolkits, to understand research partner perspectives on health needs and research priorities [49].

#### 4.1.2 Nurture Social Connection

Caregivers of a young child with CP, like parents of children with other disabilities, describe feelings of stress, depression and reduced social support [50, 51]. They also emphasise the value of connection to providers and to other parents [52]. In the present initiative, the value of fostering parent‐to‐parent interactions was exemplified in the caregiver statement, ‘Participation in research studies is a shared experience, so we [participants] should connect.’

In addition, adults with CP—especially in those who use alternative communication technologies—have an elevated risk of loneliness relative to peers without CP [53, 54]. As such, social connection—or its absence—may have implications for how people with lived experience of disability engage in research. Empowering social connections between people with CP or their caregivers via a research study design may enhance the value of research participation.

The need for social connection in research extended to clinicians and trainees, who expressed this need through learning opportunities, professional community connections and interdisciplinary and interinstitutional collaborations. For example, trainees reported that a desire to ‘want to do it [work in the field of CP]/care about it personally/[feel] personally connected]’ was a major determinant of entering and/or staying in the field. Similarly, one trainee programme administrator commented that trainees ‘struggle to see the importance of research and evidence‐based practices’ and ‘students are not always interested in paediatric research.’ For their part, clinicians rated connection subthemes, such as learning experiences, inclusion on research teams and involvement in study development, as somewhat‐to‐very‐useful facilitators of research‐clinician collaborations that were not consistently being implemented across institutions.

Researchers, who may contact a wide range of lived experience partners throughout their studies, can deploy methods to connect these lived experience partners to each other. Although strict guidelines exist for the protection of human participants in research and private health information, ‘opt‐in’ approaches allowing participants control of who, what and how information is shared. This can balance the connection with confidentiality. In addition, partnering with study participants to develop, organise and host social events can provide creative, feasible and accessible opportunities for connection. These events can nurture relationships that build trust in the researcher's ability to listen and respond to lived experience partner needs.

Researchers seeking to address these barriers can partner with trainees and programme administrators to design and promote opportunities for interdisciplinary education and training in clinical research. Programme administrators suggested capstone projects, paediatric residency programmes combining research and clinical experience, long‐term projects for students interested in research and internship programmes that incorporate research into designated student hours. Other proposed solutions from trainees included increasing awareness of opportunities through professors, conferences and posts on ‘[their] own social media pages to connect with friends and other students [who do or may be interested in CP].’

#### 4.1.3 Build Trust and Codesign Studies With Research Partners

Distrust of researchers, lack of understanding of the clinical process and fear of risk are some factors that challenge nurturing social connection among research partner groups, and especially lived experience partners [36]. Examples of these issues emerged in researcher responses (e.g., equipment that perpetuates disparities, and historical lack of trust in research, especially among underrepresented minorities). Mindful of factors that contribute to infant developmental risk [55], efforts were made in the present initiative to ensure racially, geographically and socio‐economically diverse caregiver participation. However, paediatric research in general has multiple opportunities for improvement.

Additionally, study burden is an often‐cited barrier to participation in clinical trials [36]. Consistent with this notion and other studies [56], respondents across all groups perceived that logistical difficulties, including time constraints, transportation and the proximity of studies to participants’ homes, were significant deterrents to participation. In the absence of a compelling reason to participate in research, these barriers are further magnified for families who experience mobility, language, cultural or socio‐economic challenges.

At the same time, involving families with a diverse range of needs in all phases of the research process, from planning to debriefing [35, 57], is a critical research step. Child‐friendly research sites and teams can make it easier for mothers to participate in trials [57, 58]. Parental input in trial design and study outcomes has recently been successfully implemented in clinical trials for neonates with neural insults [59], whereas family unit and siblings' involvement present new opportunities for engagement.

Establishing shared purpose around the health needs of lived experience partners, and including lived experience partners as equal partners during the planning phase of studies, can foster feelings of appreciation and value. Creative recruitment strategies such as open access screening initiatives, utilisation of patient registries and community outreach efforts enhance accessibility for people with disabilities who are typically difficult to reach [60, 61, 62].

Further, clinical trials integrated into healthcare systems provide a potential alternative to traditional approaches [63, 64]. They combine clinical research with routine care delivery [65], thereby reducing barriers to research participation for both lived experience and clinician partners. The proposed research actions described subsequently align well with such a framework.

### 4.2 Limitations and Strengths

Data collected through qualitative designs, such as discussion and open‐ended questioning, necessitates smaller sample sizes than quantitative surveys, which could be considered a limitation. However, it allows for deep exploration of beliefs, sensitive issues, relationship building and community empowerment. To fully realise the CBPR approach, feedback on developmental opportunities identified and proposed future actions is needed from the other research partner groups. Finally, representativeness of participants is essential for contextualising findings, so caution is needed generalising findings beyond the target population. Intentional efforts were made to recruit a racially, geographically and socio‐economically representative sample of caregivers of children under 3 years in Georgia, but the generalisability of these findings has not been confirmed.

## 5 Conclusions and Future Directions

Expanding the assessment of research needs and perspectives across Georgia and throughout the United States will continue to inform CP initiatives, interinstitutional collaborations and communication pathways. Further identifying partner perspectives on facilitators and barriers to CP clinical care is critical for improving health outcomes and eliminating health disparities in Georgia through community‐focused research that can improve outcomes in CP across the lifespan.

## Author Contributions

**Melissa M. Murphy:** conceptualization, data curation, formal analysis, methodology, writing – original draft. **Gavin T. Colquitt:** conceptualization, methodology, writing – review and editing. **Paige S. Ryals:** conceptualization, data curation, formal analysis, methodology, writing – review and editing. **Katie Shin:** data curation, formal analysis, writing – review and editing. **William C. Kjeldsen:** formal analysis. **Allison McIntyre:** conceptualization, writing – review and editing. **Sydni V. W. Whitten:** conceptualization, data curation, writing – review and editing. **Christopher M. Modlesky:** conceptualization, funding acquisition, resources, writing – review and editing. **Nathalie L. Maitre:** conceptualization, funding acquisition, resources, writing – review and editing.

## Disclosure

The funders had no role in the design of the study; in the collection, analyses or interpretation of data; in the writing of the manuscript; or in the decision to publish the results.

## Ethics Statement

The researcher project was deemed as ‘not research involving human subjects’ by the University of Georgia Human Subjects Office (PROJECT00007325). Ethical review and approval were waived for this study due to the projects being quality improvement initiatives, and so deemed as not ‘human subjects research’ by the Emory Institutional Review Board. Participant consent was obtained verbally as no identifiable data were collected, and the project was for quality improvement purposes.

## Consent

The authors have nothing to report.

## Conflicts of Interest

The authors declare no conflicts of interest.

## Supporting information

Supplemental CP Research clean copy for publication.

## Data Availability

The data sets analysed during this study are available from the corresponding author upon reasonable request.

## Appendix A

Clinician Survey Questions Regarding Clinical Participation in Research**

***These questions were posed as part of a longer survey of questions about barriers and facilitators of healthcare access. Results from the other questions are discussed in a separate manuscript*.

How well does your programme implement the following ‘practices’ in its efforts to help researchers and clinical practitioners collaborate at your institution or practice? On a scale of 1–100 (not well at all ‐> very well).

- Clinicians play an active role in the development of research studies

- Clinicians play an active role in the administration of interventions and/or assessments of research studies

- Clinicians are able to easily refer a patient to actively recruiting studies

- Clinicians regularly receive updates (e.g., study result summary) from researchers when studies have been completed

- Clinicians are compensated for their time engaged in research studies

- Clinicians are trained about comparative effectiveness research and its benefits for clinical care

- ‘add your own’

How much do you think the following practices would be helpful to allow CP researchers to collaborate more effectively with clinicians moving forward? Scale 1–100 (not at all helpful ‐> very helpful).

- Researchers could involve CP clinicians more in the development of a research study or aim

- Researchers could design more research studies that seamlessly integrate within standard clinical care processes

- Researchers could provide updates to clinicians in the format of short briefs when a research study has been completed

- Researchers could host a more formal learning experience for newly published research (e.g., CEU credit courses, lunch and learn webinar and grand rounds)

- Clinicians could be an active part of the research team in performing tests, assessments and/or interventions

- A platform that hosts currently active studies looking for the recruitment of participants

- A platform that hosts study updates for completed studies

- Compensating clinicians effort on research studies

- ‘add your own’

How much do you think the following are or would be barriers to preventing clinician collaboration with CP researchers? Scale 1–100 (not a barrier at all ‐> very much a barrier).

- Clinicians do not have enough time to participate in research activities

- Clinicians are not paid enough to participate in research activities

- Clinicians are not interested in being involved in research activities

- Clinicians do not know enough about research designs or its benefits for clinical care

- Clinicians do not have access to information about research studies being conducted

- Clinicians do not have access to CP researchers

- Clinicians do not have enough training to participate effectively in research activities

- ‘add your own’

Is there anything else that you would like to add about clinician participation in research? Open Ended

Training and Education

- How important are the following with regard to maintaining a pathway for upcoming clinicians who treat CP? Scale 1–100 (not at all important ‐> very important).

- Trainees are provided mentors within the field

- Trainees are exposed to multiple pathways or areas of interest throughout training

- Trainees are provided help/ways to fund their training

- Trainee programmes engage or promote relationships across institutions

- Clear and consistent communication from programme administration regarding expectations of training

- ‘add your own’

How frequently does your practice or institution do the following in terms of maintaining a pipeline of upcoming clinicians who treat CP?  On a scale of 1–100 (none of the time ‐> all the time).

- Trainees are provided mentors within the field

- Trainees are exposed to multiple pathways or areas of interest throughout training

- Trainees are provided help/ways to fund their training

- Relationships with other institutions and programmes

- Clear and consistent communication from the administration regarding expectations of training

- ‘add your own’

How much do you think the following are or would be barriers to trainees entering or remaining in the field of caring for patients with CP? Scale 1–100 (not a barrier at all ‐> very much a barrier).

- Perceived time commitment of this training

- Perceived complexity of treating patients with CP

- Trainees do not have access to education/training in treating patients with CP

- Trainees are not aware of opportunities to work with patients with CP

- Training is too expensive

- ‘Add your own’
