# Supplementary material for: Synergies, Discrepancies, and Action Priorities: A Statewide Engagement Study to Strengthen Clinical Research in Cerebral Palsy
Source: Health Expect. 2025 Apr 24;28(3):e70257. doi: 10.1111/hex.70257 (PMC12022003; doi:10.1111/hex.70257)
Supplement: Supplementary file 1 — Supplemental CP Research clean copy for publication. [file HEX-28-e70257-s001.docx]

Supplemental

| **Table S1.** Partner group engagement methodology adapted from World Café Community Foundation (2015) World Café Guidelines: *Seven Design Principles* | | |
| --- | --- | --- |
| Step | Recommended Design Principle | CP Application |
| **Stage** | **Select partner groups.** Determine who should take part in the conversation. Likely there is more than 1 group for a given issue. In which case, select the group upon which you want to focus for each specific conversation. | Five groups were identified: (1) Applied, clinical, and basic science researchers in the field of CP, (2) caregivers of a young child with CP, (3) clinicians specialized in CP early detection and high-risk infant follow up, (4) trainees participating in CP research programs and/or clinical care, and (5) university training program administrators. |
|  | **Set the context.** Select a context that brings members of the partner groups together. | Caregiver, researcher, clinician, and trainee perspectives were collected at CP-focused events taking place from April 2023-July 2023. Program Administrator perspectives were collected during individually scheduled interviews between November 2023-January 2024. |
|  | **Determine what themes are most relevant and what questions matter.** Identify the topics or themes to discuss that reflect real-life concerns of the group. Craft compelling questions that foster discussion. | The theme “barriers and facilitators of research participation for partner groups” was determined based upon input from informal discussions with CP researchers planning the 2023 Research Symposium on the State of CP. Responses from this group guided questions for other groups. |
|  | **Plan the forum.** Using information from Steps 2&3, set a timeframe and discussion round pacing. | Focus group format followed the World Café approach to engagement. |
| **Implement** | **Encourage everyone’s contribution.** Use strategies that ensure active participation of all participants. | Prompts were posed by the session facilitator with time allowed for small group discussion. A “host” was assigned for each small group to keep pace and ensure everyone had an opportunity to share. |
|  | **Connect Diverse Perspectives.** Mix up the participants between discussion rounds or encourage whole group sharing to encourage interactions others and shared perspectives. | Depending upon time allowed for the focus group, participants switched groups between rounds. All groups participated in the whole group discussion of each prompt. Additional responses sent via email after the sessions were encouraged. |
|  | **Listen together for patterns and insights.** Encourage shared listening and attention to themes, patterns, and insights. | Two facilitators circulated during the rounds to listen and encourage discussion. |
|  | **Share collective discoveries.** Make discussion content visible to the whole group by inviting reflection and sharing with the larger group. | Following each discussion round, the facilitators led the whole group discussion asking each table to share aloud the themes and insights emerging from the table conversation. |
| **Analyze** | **Summarize the responses.** Examine discussion content looking for common themes and subthemes (i.e., “opportunities”) across responses and discussion rounds. | After the conference, members of the research team transcribed, reviewed, and coded the responses collected during the discussions. |
| **Circle back** | **Seek feedback from participants** on opportunities identified and elicit proposed future actions. | A summary of the researcher results was shared with the *2023 Research Symposium on the State of CP* planning committee for feedback on themes and proposed future actions. These results informed the clinician survey and were combined with responses from other partner groups to prioritize future actions. Results of the whole initiative will be shared with all partner groups for feedback and to arrive at a consensus for priority actions for CP research. |

**Table S2.** CP Researcher World Café Questions

| What has gone well in your efforts to establish, maintain, or contribute to a high-quality program of research in CP at your institution? What needs improvement? |
| --- |
| What are some ways that researchers and clinical practitioners can collaborate more effectively on CP-focused initiatives?  What are barriers to trainees (post doc/grad students or clinical students/interns/fellows) entering or remaining the field of CP research for your institution and GA as a whole? What are possible solutions to these barriers? |
| What does it mean to you to when you hear “patient/family engagement in research design and priorities”?  What does your group do well to engaging patients/families in designing studies and setting CP research priorities?  What would need change for you to engage patients/families more fully in designing research and setting priorities? |
| What does your group do well in your efforts to recruit and retain patient/families in your CP research? What are the barriers to patient/family recruitment in GA?  If you were to design a research registry for patients/families with CP in Georgia, what would it include? |

**Table S3.** CP CaregiverWorld Café Discussion Prompt

| Pretend you have learned about a research study that is actively recruiting participants. This study is looking at a topic that will help us learn more about cerebral palsy and/or the possible treatments or interventions that can help people with cerebral palsy. This study involves both you and your child with cerebral palsy participating, but it does NOT involve any invasive procedures (for example, medications or lab work).  *What are some of the things that you would want to know or consider before deciding if you could/would want to participate?* |
| --- |

**Table S4.** Trainee and Program Administrator Questions

| Trainees: What are barriers to you entering or remaining in the field of CP for your institution and GA as a whole? What are possible solutions to these barriers? |
| --- |
| Program Administrators: (1) What you see as your school or department’s needs for student trainee opportunities, especially related to clinical care and research for cerebral palsy and other developmental disabilities. (2) What are the barriers to students entering or remaining in the field of CP for your institution and GA as a whole? What are possible solutions to these barriers? |

| **Table S5.** Examples of *Involvement* theme and subtheme responses related to barriers and facilitators of CP research (researchers, trainees, program administrators) and considerations for participating in research (caregivers) | | |
| --- | --- | --- |
| **Involvement** | | |
| **S** | *awareness of opportunities* | **Researcher:** “Lack of knowledge about what is available regarding research.” “marketing needs improvement” “dissemination of research/access broadly is not good” “we [lab group] leverage multimedia platforms (soc [media], web, clinic, fliers, word of mouth)” |
|  |  | **Caregiver:** “How to find such studies/eligibility.” |
|  |  | **Trainee:** “Need for increasing awareness about these opportunities: conferences, opportunities in research, advocacy.” “Students have to find a connection with a professor to find out about the opportunities”  **Program Admin:** Not observed. |
| **S** | *logistical burden to access to opportunities* | **Researcher:** “Travel assistance, especially for more severe impairment.” “Transportation to and from lab.” |
|  |  | **Caregiver:** “Time commitment involved: how much time is spent actively participating, at home commitments, what happens with the research.” “Length/frequency/location/ cost [of participation]” |
|  |  | **Trainee:** “Families not having funds to pay for early intervention services”  **Program Admin:** “Location of internship will influence where graduates stay (will likely stay at internship location if offered job)” |
| **S** | *attitudes & perspectives^a^* | **Researcher:** General desire [among researchers & clinicians] to progress for a common goal [to improve outcomes for people with CP]” **“** |
|  |  | **Caregiver:** Not observed |
|  |  | **Trainee:** “Preconceived ideas about CP and what it is.”  **Program Admin: “**Students have trouble applying knowledge/content to real world contexts” “Students struggle to see the importance of research and evidence-based practices” “Students are not always interested in pediatric research” |
| **D** | *informational needs* | **Researchers:** “Increase participant investment in research by providing clear information regarding plan from the start.” “Parent education.” “Clear dissemination of research focus as opposed to therapy focus.” |
|  |  | **Caregiver:** “Parents would like to know how kids have benefited from the research, even if just anecdotally.” “Access to a write up of individual results or developmental assessments would be nice.” "Making sure there won't be any side effects or interference with current therapies" “presenting benefits foremost and highlighting time commitments and scheduling flexibility” “What are the potential benefits for my child” |
|  |  | **Trainee:** Not observed.  **Program Admin**: “Explanation of research method.” “Preparation for project proposals (including funding conversations and other requirements for pitching a program).” |
| *Note*. S = synergy; D = discrepancy | | |

| **Table S6.** Examples of *Connection* theme and subtheme responses related to barriers and facilitators of CP research (researchers, trainees, program administrators) and considerations for participating in research (caregivers) | | |
| --- | --- | --- |
| **Connection** | | |
| **S** | Professional community connection & development | **Researcher:** “State-wide efforts towards retention of researchers, clinicians, etc: investment of money and time into high school, colleges, research, and beyond.” |
|  |  | **Caregiver:** Not observed. |
|  |  | **Trainee:** “Students/trainees having their own conversations with other students, colleagues.”  **Program Admin:** “Create professional development opportunities to attract stronger students (seminars or workshops).” |
| **S** | Interdisciplinary & Interinstitutional collaborations | **Researcher:** “Collaborations between clinicians/physicians at academic institutions.” “Sharing research/funding across several institutions.” |
|  |  | **Caregiver:** Not observed. |
|  |  | **Trainee:** “Encouraging relationships across disciplines between students and trainees.”  **Program Admin:** “Opportunities for students to gain interdisciplinary experience” “Experiences mixing clinical and research experiences.” |
| **S** | Educational and/or training initiatives | **Researcher:** “Offering CEUs to clinicians for assistance in research.” “Internship programs for resident students/clinicians with researchers.” |
|  |  | **Caregiver:** Not observed. |
|  |  | **Trainee:** “Not having the infrastructure to provide any kind of support or education/attention toward CP at some institutions or countries throughout the world”  **Program Admin:** “Placements in pediatrics for capstone projects.” “The lab provides opportunities for students to see research/application into real practice.” “Creation of funded projects for students interested in research” |
| **-** | Involvement of K-12 partnerships^a^ | **Researcher:** “Integrated research with school system.” “Implementing the research in school-based programs.” “School-based data collection & special Olympics.” |
| **D** | *CP community connection* | **Researcher:** “Social interactions between participants' families participating in research.” “Increase knowledge of CP community through exposure to individuals with CP.” |
|  |  | **Caregivers:** “Connecting with other people doing it, experienced it.” “Participation in research studies are a shared experience so we should connect.” |
|  |  | **Trainee**: “Need for building partnership between family and communities to be able to continue connections throughout the lifespan.”  **Program Admin***:* Not observed |
| **D** | *Pathways for bidirectional communication among stakeholders* | **Researcher: “**Collaborations between researchers and clinicians.” “Incorporating patient-family lived experiences into current research to strengthen research direction.” “Needs work: communication from researchers back to clinicians and between medical providers and school-based providers.” |
|  |  | **Caregivers:** “Maybe we could have a meeting ground once a month for research participants” |
|  |  | **Trainee:** “Lack of infrastructure/pathways across age groups as children grow up to be able to continue communication and involvement in CP Community and research.” “Central platform and portal.”  **Program Admin***:* “Create professional development opportunities to attract stronger students (seminars or workshops)” |
| *Note*. S = synergy; D = discrepancy; ^a^Noted only among researcher group | | |

| **Table S7.** Examples of *Research Approach* theme and subtheme responses related to barriers and facilitators of CP research (researchers, trainees, program administrators) and considerations for participating in research (caregivers) | | |
| --- | --- | --- |
| **Research Approach** | | |
| **S** | *Participant experience during research recruitment/ participation* | **Researcher**: “Parent engagement and satisfaction: trust (once they're in the door, they stay).” “Give the parent reason to want to come back (benefits wise).” |
|  |  | **Caregivers:** "... did participate in a research study during Covid ... using telehealth and it worked well... it helped that there were clear instructions." |
|  |  | **Trainee/Program Admin***:* Not observed |
| **D** | Ethical research & equitable representation in research | **Researcher: “**Retaining diverse populations.” [addressing] SES [barriers]” “Communication to rural areas.” “More people with CP should do research.” “Clear tangible benefits for stakeholders” “Equipment that perpetuates disparities (e.g., EEG caps that require wetting hair)” |
|  |  | **Caregivers:** Not observed |
|  |  | **Trainee/Program Admin***:* Not observed |
|  |  | **Clinicians:** Not rated |
| **-** | Research design and/or process *^a^* | **Researcher:** “home-based intervention to [improve/support/enable] parent compliance and consistency” “Standardizing information collection methods.” “Integrate play [into research protocols & assessment testing days]” |
| **-** | Study information data elements *^a^* | **Researcher:** “Benefit, expectations, outcomes given to family.” “Testimonies of participants/rating system [to rate studies].” |
| **-** | Quality Control *^a^* | **Researcher:** “More tightly controlled data collection.” “Data quality/test design.” “Screening process is inefficient when based on EMR/charts.” |
| **-** | Potential participant data elements*^a^* | **Researcher:** “Clear diagnosis, med history, therapeutic history, previous imaging, other research participation, chief complaints, valid contact info, goals, visual representation of participant functional abilities.” |
| **-** | Strategies for increasing recruitment*^a^* | **Researcher:** “Flyers in clinics/schools.” “Sharing contact lists between labs.” “Registry would help.” |
| *Note*. S = synergy; D = discrepancy; *^a.^Subtheme observed only among researcher group.* | | |

| **Table S8.** Examples of *Funding* theme and subtheme responses related to barriers and facilitators of CP research (researchers, trainees, program administrators) and considerations for participating in research (caregivers) | | |
| --- | --- | --- |
| **Funding** | | |
| **S** | *Clinician/trainee support* | **Researcher:** “Funded post-doc opportunities.” “Finding [funding] solution for state clinician participation.” |
|  |  | **Caregiver**. Not observed. |
|  |  | **Trainee:** “Need for financial, transportation, housing support to be able to participate/attend these opportunities that may take place at other institutions in particular.” |
|  |  | **Program Admin:** “Lack of financial assistance during internships.” |
| **-** | *Research process or infrastructure*^a^ | **Researcher**: “Funding” “Funding from counties (rural especially and low SES) [to support research initiatives in those areas]” |
| **D** | *Family compensation* | **Researcher**: “Compensation to the individual and families for their time and their expertise.” “Payments to the caregivers for their services as well; designing better studies.” |
|  |  | **Caregiver:**  Not observed. |
|  |  | **Trainee/Program Admin**: Not observed. |
| *Note*. S = synergy; D = discrepancy;  ^a^Noted only among researcher group. | | |
